# Tribological Characterization of Electrical Discharge Machined Surfaces for AISI 304L

**DOI:** 10.3390/ma15031028

**Published:** 2022-01-28

**Authors:** Muhammad Adnan, Waqar Qureshi, Muhammad Umer, Daniele Botto

**Affiliations:** 1 Mechanical Engineering Department, University of Engineering & Technology Taxila, Taxila 47050, Pakistan; muhammad.adnan@students.uettaxila.edu.pk (M.A.); waqar.ahmed@uettaxila.edu.pk (W.Q.); 2 Mechanical Engineering Department, Institute of Space Technology Islamabad, Islamabad 44000, Pakistan; muhammad.umer@ist.edu.pk; 3 Department of Mechanical and Aerospace Engineering, Politecnico di Torino, 10129 Torino, Italy

**Keywords:** boundary lubrication, friction, surface roughness, SEM, abrasive wear

## Abstract

Surface treatments are normally carried out after machining. Surface treatment is a costly and time-consuming process. Hence, it makes sense to reduce the requirement of surface treatment as much as possible. Electrical Discharge Machining (EDM) is a frequently used machining process. EDM produces a recast layer on the surface of machined components. The tribological performance of this recast layer is not very well understood. The properties of the recast layer formed as a result of EDM depend upon the discharge current, electrodes and dielectrics. This work aims to study the effects of each on the tribological performance — in terms of the wear depth, friction coefficient, friction force and contact surface temperature of recast layers. Subsequent improvement in the quality of surfaces will significantly reduce the cost and time required to treat surfaces after machining. Hence, various combinations of discharge current, dielectrics and electrodes have been used to characterize and deduce their effects. The tribo-tests are performed in the boundary lubrication regime under pin-on-disc configuration to analyze sliding friction, contact surface temperature and the wear of the recast layers formed on AISI 304L. The surface morphology of the test pins has been performed by Scanning Electron Microscopy (SEM) before and after the tests. The results show that indeed it is possible to control the tribological performance of the recast layers by varying EDM parameters. This approach promises to be a useful methodology to improve the tribological performance of the layers formed after EDM and reduce the time and costs required for surface treatments post machining.

## 1. Introduction

Machined components are extensively used in applications involving contact. In such cases, tribological properties become very important. Improved tribological properties result in extended service life, higher efficiency, the reliability of the mechanical systems and also conserve energy [1,2,3]. Various surface treatments methods including *surface coating, ion implantation and surface texturing* are used to alter the tribological properties of machined surfaces. A brief overview of these methods is as follows:

*Surface coating* modifies the tribological properties of the surfaces and protects them from the environment. Choudhary et al. [4] investigated the crystalline and adherent coating by reactive magnetron sputtering on stainless steel substrate. Pereira et al. [5] found that, at high temperature, the wear rate of stainless steel surfaces is reduced due to coating. Atul et al. [6] applied surface mechanical attrition treatment on AISI 304L surfaces for increasing the corrosion resistance. Tyagi et al. [7] used WS_2_ and Cu powder mixture for solid lubrication and enhanced tribological performance. Coating is very important in additively manufactured components, in which dry friction contacts play a fundamental role in fatigue failures [8,9]. Li et al. [10,11] studied frictional contact over the physical contacted surfaces. Another surface treatment method used for alteration of tribological performance is *ion implantation*. Foerster et al. [12] studied the nitrogen ion beam process of stainless steel surfaces. López et al. [13] analyzed the corrosion-erosion synergism of AISI 304L affected by nitrogen implantation. Yang et al. determined that when AISI 304 is effectively nitrided at cathodic and floating potentials, its tribological performance improves [14]. Xie et al. [15] studied the tribological properties of titanium-ion-implanted surfaces. *Laser texturing* has also been used in the recent years for the improvement of the tribological performance of different materials [16,17,18,19,20,21]. Wang et al. [22] studied micro-groove positioning, friction coefficients and wear rates of textured surfaces. Wos et al. [23] studied the surface texturing of sliding elements. Saada et al. [24] investigated that wear and tribocorrosion are reduced by nanoscale surface peening when a nanocrystalline surface is applied over AISI 304L. Vishnoi et al. [25] gave detailed information about surface texturing to enhance tribological performance.

All these surface treatment processes are carried out after the machining of the surfaces. They require time, high capital cost and the requirement of sophisticated equipment and the specific environment [26]. The work at hand aims to reduce the time and costs required for post-machining surface treatment processes. A better tribological behavior is sought by varying the EDM parameters during machining at no additional cost.

EDM is a frequently used machining process for all electro-conductive materials. It finds applications in the aerospace, automotive, surgical instrument and mold industries [27]. EDM uses serially applied discharges between a work-piece and an electrode. The melted material solidifies quickly and produces a recast layer on the machined surface [28]. This recast layer has different metallurgical structures and properties as compared to the base material [29]. The recast layer reduces the fatigue strength and deteriorates the surface integrity [30]. Many studies have been conducted to investigate and improve the characteristics of the recast layer. Xavior et al. [31] investigated the effects of the recast layer on the strength characteristics of the Inconel 718 alloy. The properties of the recast layer formed as a result of EDM were found to depend upon the discharge current, electrodes and dielectrics. Ojha et al. [32] found that the discharge current significantly affects the material removal rate, dimensional tolerance and surface roughness. Rahman et al. [33] concluded that a product of long pulse duration and high discharge current causes rough surfaces. Leao et al. [34] concluded that the work-piece surface roughness / integrity is dependent on the type of dielectric fluid used. It also affects factors such as productivity, quality, health, safety and environment. Chakraborty et al. [35] determined that distilled water as a dielectric results in a better surface finish and poor machining accuracy. They also found that surface roughness produced with deionised water is generally lower than that with hydrocarbon oils.

Very few studies are available on tribological characteristics of recast layers produced as a result of EDM. Ekmekci et al. [36] studied the wear resistance of EDM-ed surfaces. Their main parameters of interest were pulse duration and tool electrode only. R. Prathipati et al. [37] investigated the wear resistance of AISI 316L machined with wire EDM. However, only electrical parameters of EDM were altered with one type of electrode and dielectric. Due to little information on tribological performance of recast layers [36,37], a more in-depth study is warranted.

The current work aims to characterize the tribological properties of recast layers formed. A portable surface roughness tester was used for the surface profilometry. EDS analysis was performed to acquire the chemical composition of the recast layers. The tribological testing was performed using pin-on-disc configuration at room temperature in the boundary lubrication regime. Surface defects and wear scar surfaces were examined by scanning electron microscopy. A digital electronic balance was used to measure the mass of samples before and after tribo-tests to calculate the weight loss. Finally, the analysis and discussions have been made.

## 2. Materials and Methods

Experimental work involved specimens preparation through conventional machining and EDM. This step involved the specimens’ characterization by tribo-tests and Scanning Electron Microscopy (SEM). Each step of the experimental activity is discussed separately. From here on, *the combination of electrode and dielectric* means the recast layer produced by the respective combinations.

### 2.1. Specimens Preparation and EDM

AISI 304L austenitic stainless steel was selected as a pin specimen. AISI 304L is extensively used in the oil industry, chemical industry and manufacturing. It is also used for making components such as pre-heater tubing, primary piping, coolant pumps, reactor cores, steam dryers and some parts of the turbines in the nuclear industry. These parts are often subjected to wear. Spectrometry of the specimen material for determining the chemical composition was performed by Oxford X-MET 5000 Handheld XRF Analyzer, which is shown in Table 1.

**Figure 1:**
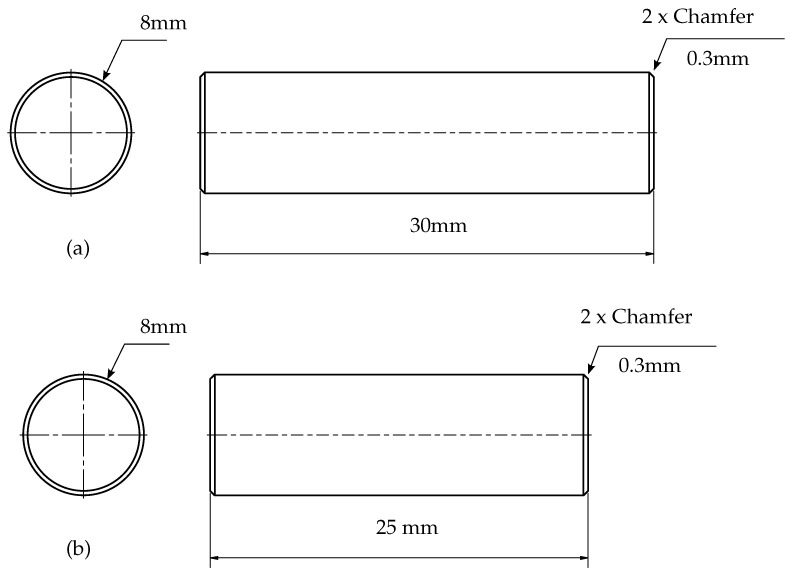
Specimen dimensions (**a**) before EDM, (**b**) after EDM.

An AISI 304L rod was machined on the conventional lathe machine BANKA 43 to obtain the diameter of 8 mm and a length of 30 mm of each specimen, as shown in Figure 1.

**Figure 2:**
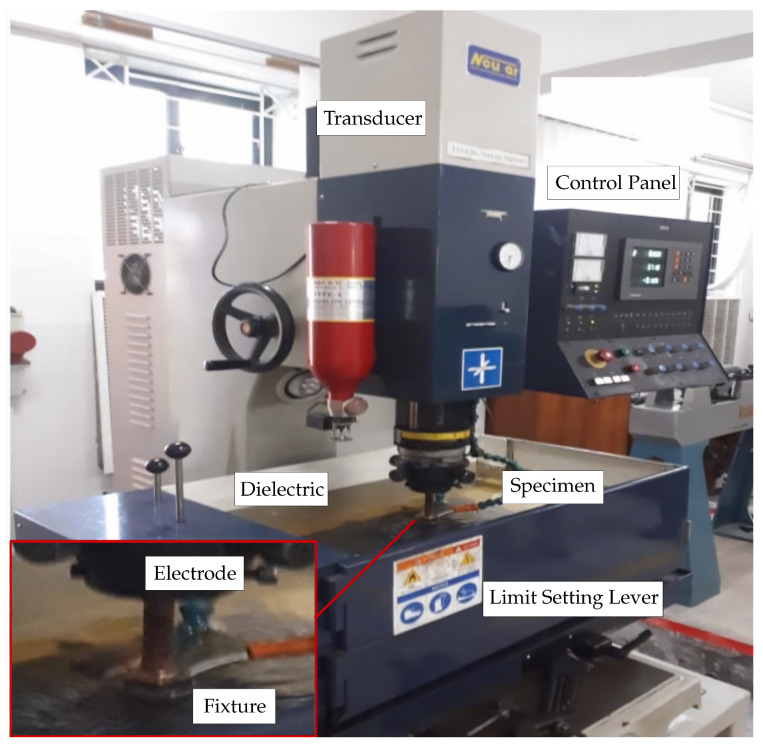
Experimental setup for EDM.

Copper and graphite electrodes of diameter 20 mm and length 300 mm were used. A fixture was made, shown in Figure 2, to strongly clamp the specimens during EDM process. The machining was performed using NEUAR Sinking EDM (Figure 2).

During EDM, copper and graphite electrodes were used with paraffin oil and distilled water as dielectric media. After each test, the electrode was ground with sandpaper to remove surface irregularities. These combinations of electrode materials and dielectric media are commonly used in the molding industry. EDM with distilled water was performed in a separately made water tank to avoid the contamination of distilled water with paraffin oil. Many combinations of EDM parameters were possible, and it resulted in difficulties in the experimental design. However, the effects of these parameters on surface characterization have been elaborated in literature. Therefore, the experimental conditions were chosen which produced significant changes in surface roughness, chemical composition and surface morphology, as shown in Table 2. Discharge current was varied while keeping all other electrical parameters constant to machine in a factorial order. Twenty samples were prepared, which were already machined on the conventional lathe machine to reduce the specimen length to 25 mm.

### 2.2. Tribological Testing of Recast Layers

**Figure 3:**
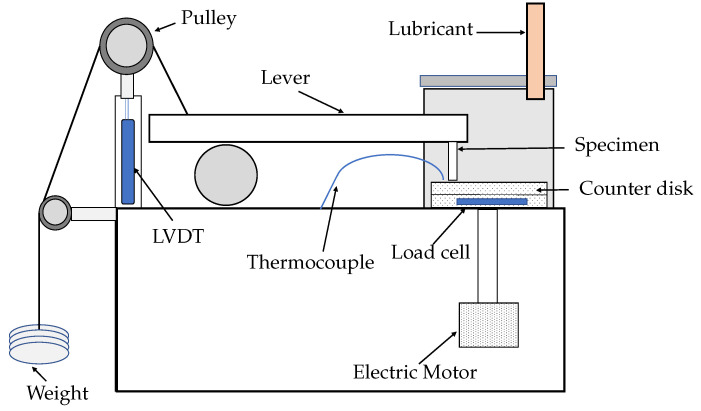
Pin-on-disk tester - schematic.

The experiments were performed using pin-on-disc tester K93900 (Bohemia, NY 11716, USA), whose schematic is shown in Figure 3. The stationary cylindrical specimen slid against a rotating counter-disk of diameter 165 mm and thickness 8 mm. The material of the disk was surface-treated EN 31 steel, which is harder than the pin of AISI 304L. The chemical composition of the disk is given in Table 3.

**Figure 4:**
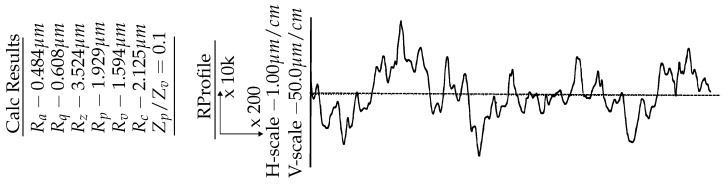
Surface profile of the counter-disk.

The roughness of the disk was measured using a portable roughness tester, Mitutoyo SJ-410 (Aurora, IL 60502, USA). Its average value was found to be $R_{a}=0.484$ μm, as presented in Figure 4. One disk was used for all the tests. After each test, the disk was ground with sandpaper to the surface roughness of $R_{a}=0.484$ μm. All tribo-tests were performed at room temperature under oil lubrication conditions. SuperMatic 4T SAE 10W-40 (Chevron Pakistan Limited, Karachi, Pakistan) was used as a lubricant for tribological testing, and its properties are given in Table 4.

Many experiments were conducted to choose the experimental conditions. It was attempted to obtain a measurable and suitable wear section after tribological testing. The applied process parameters were based on the indentation produced on recast layers, as shown in Table 5.

After setting all parameters and sensor calibrations, the test was initiated. The wear depth, frictional force and contact surface temperature were measured by linear variable differential transducer (LVDT, least count 0.1 μm), beam type load cell and k-type thermocouple, respectively. The data acquisition system of the tribotester displayed and stored the sensors’ data. The very thin recast layer made it quite difficult to repeat the tests without reaching the substrate. However, similar tests on multiple samples do give a fair assessment. Weight loss was calculated by the difference in masses before and after the tests $\left(M_{1}-M_{2}\right)$. Specimens were cleaned using acetone and ultrasonic tank to remove paraffin oil. The mass of each specimen was measured using a digital electronic balance with an accuracy of 0.01 mg. The measurements were repeated three times for each sample to determine the average weight loss.

### 2.3. Profilometry of Recast Layers

Surface roughness of the recast layers was measured using a portable roughness tester, Mitutoyo SJ-410. The average surface roughness was calculated from three measurements.

### 2.4. Surface Morphology of Recast Layers

Specimens were prepared to find out the properties of the recast layers after EDM. Samples were cleaned by the ultrasonic bath to obtain the clear images. These were placed on the turntable of VEGA3 TESCAN (Brno-Kohoutovice, 62300 Brno, Czechia) before the tribo-tests. Surface defects were examined during surface morphology that affected tribological performance later. Energy dispersive spectrographs were acquired to study the chemical composition of recast layers. After the tribological testing, specimens were again prepared for examination of the worn surface by SEM. Worn surface characteristics were examined by taking images at 500× magnification.

### 2.5. Cross-Sectional Investigation of Recast Layers

For cross-sectional investigation, specimens were cut perpendicular to EDM-ed surface. They were embedded in cold resin epoxy, followed by grinding (2000 grit sandpaper) and polishing. The samples were etched with a solution of hydrochloric acid (119 mL), nitric acid (12 mL) and distilled water (119 mL) at 20 °C for 15 s. Optical microscope OLYMPUS BX51 (Shinjuku-ku, Tokyo 163-0914, Japan) and image processing technique ImageJ were used for recast layer thickness measurement at 50× magnification and 20 μm resolution. The measurements were repeated thrice for each sample to determine the average recast layer thickness. Hardness of the recast layers was determined by HBRVU-187.5 Brinell, Rockwell & Vickers optical hardness testers (Sino Age Development Technology, Beijing, China). The average hardness was calculated from three measurements. The EDS of the recast layers at the surface gave the composition of recast layers. It was observed that the EDS of the cross-sectional area also gave quite similar trends owing to the very small thickness of recast layers. Hence, only results for surface analysis were considered.

## 3. Results and Discussion

### 3.1. Surface Characterization Results

Discharge current was varied from 4.5 A to 15 A for two types of electrodes and dielectrics. Variations in EDM parameters produced different surface characteristics (surface roughness, chemical composition and surface morphology), which are discussed below.

#### 3.1.1. Surface Roughness of Recast Layers

**Figure 5:**
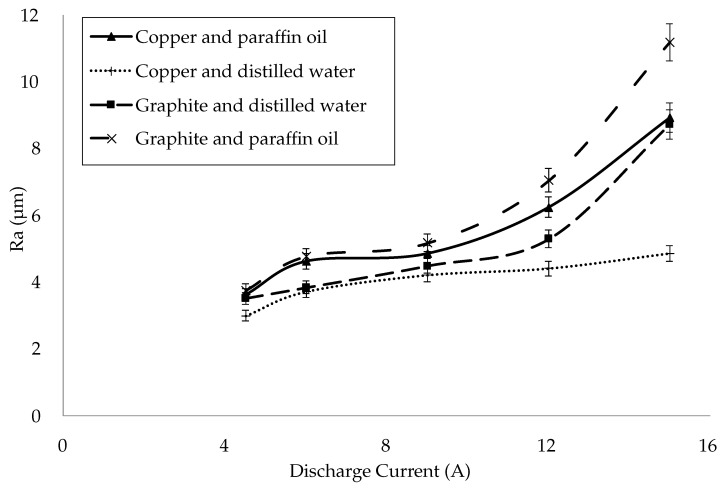
Surface roughness of EDM-ed specimens.

The effect of discharge current on surface roughness was analyzed by varying it from 4.5 A to 15 A. For the electrode-dielectric combination of Copper–Distilled water, the Ra of the recast layer was 2.987 µm at 4.5 A. The Ra value increased to 4.849 µm at 15 A for the same configuration. Similarly, the Ra values for Graphite–Distilled water increased from 3.509 to 8.719 µm for the same ranges of currents. Copper–Paraffin oil and Graphite–Paraffin oil combinations also showed similar trends. Overall, Ra values increased almost linearly with an increase in the discharge current. These results have been presented in Figure 5 and Table 6.

The highest Ra value (11.184 μm) of the recast layer was obtained for the Graphite–Paraffin oil combination followed by the Copper–Paraffin Oil (8.918 μm), Graphite–Distilled water (8.719 μm) and Copper–Distilled water (4.849 μm) combinations. The Copper–Distilled water combination produced the smoothest recast layer. The recast layers produced by Graphite–Distilled water, Copper–Paraffin oil and Graphite–Paraffin oil had 28%, 40% and 58% more surface roughness, respectively, as compared to that produced by Copper–Distilled water. The reason for these observations was the formation of carbides by the decomposition of the graphite electrode and the cracking of the paraffin oil that increased the surface roughness. The paraffin oil dielectric and graphite electrode produced high-intensity sparks, resulting in a rougher surface. The surface roughness was more sensitive to the paraffin oil as compared to distilled water for both electrodes. Similarly, graphite influenced the surface roughness more than copper for both dielectric media. Moreover, the high discharge energy due to the high discharge current enhanced the melting and vaporization of the specimen material. It generated larger and deeper craters, resulting in a rougher surface. These outcomes were also supported by the studies of [32,33]. The surface roughness was a resulting parameter after electrical discharge machining. It varied with the variation in the discharge current for each combination of electrode and dielectric.

#### 3.1.2. Chemical Composition of Recast Layers

**Figure 6:**
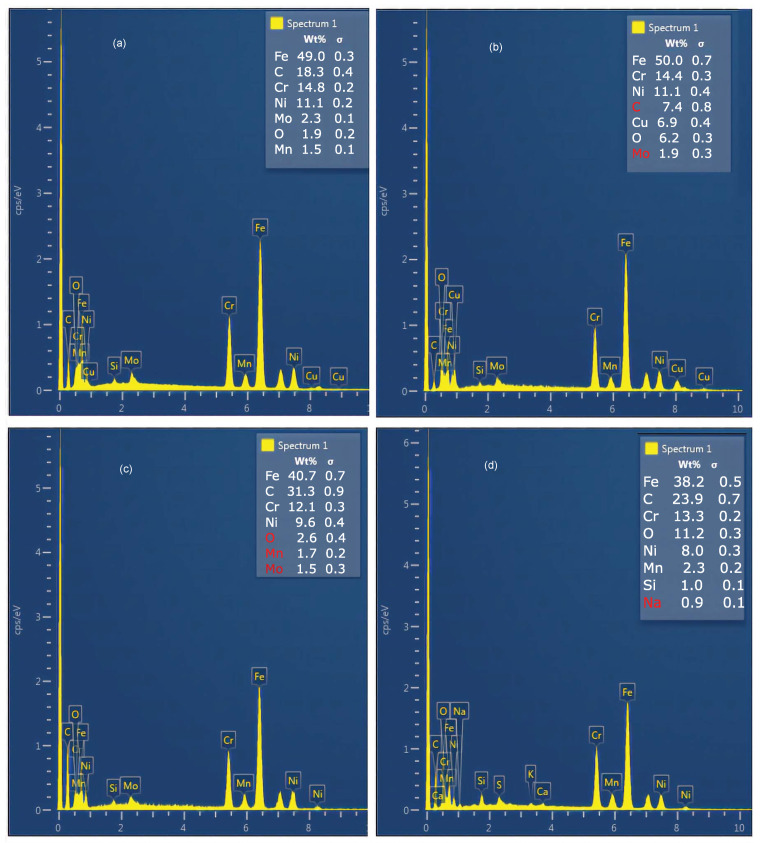
Energy dispersive spectrograph (EDS) of (**a**) copper electrode and paraffin oil dielectric, (**b**) copper electrode and distilled water dielectric, (**c**) graphite electrode and paraffin oil dielectric, and (**d**) graphite electrode and distilled water dielectric.

Since the radius of the surface to be measured was just 0.4 cm, it was decided to pick six to nine different regions representative of surface characteristics such as color, globules, appendages and material deposition. Finally, an average has been taken for all the elements, as shown in Figure 6. The EDS analysis of the machined specimens revealed that oxides were formed on the recast layer for the Copper–Distilled water combination.

Iron (Fe), nickel (Ni), chromium (Cr) and molybdenum (Mo) were reduced in the recast layers as compared to the base material. Copper (Cu) and oxygen (O) were added (6.84% and 6.2% of weight, respectively). The addition of copper and oxygen could be because of the chemical reaction between the water and copper. In case of the graphite electrode and the distilled water dielectric, carbon content (C) was added (23.88% weight) with a maximum reduction in iron and nickel. The combination of the copper electrode and the paraffin oil dielectric added 18.28% weight of carbon in the recast layer. The oxides and copper addition were minute, whereas iron loss was significant. The alloying elements depleted from the recast layers were compensated by electrodes material and dielectric media during EDM. It was observed that the combination of the graphite electrode and the paraffin oil dielectric had a significant amount of carbon (31.28% weight) diffused in the recast layer. It was due to the decomposition of the graphite and more cracking of the paraffin oil. A similar phenomenon was reported by [38]. The loss of iron was the highest, followed by chromium and, lastly, nickel. Researchers also concluded that, using an oil dielectric for steel specimens, carbon contents in the recast layers were increased after machining. Carbon resulting from the pyrolysis of the dielectric was transferred towards the molten specimen material, where alloying took place. The carbon formed additional carbides (Fe${}_{3}$C) [39]. The results in the present study were in agreement with this observation. EDS analysis indicated low-intensity copper peaks and high-intensity carbon peaks when copper and graphite were used as electrodes, respectively. Distilled water as a dielectric produced recast layers with less carbon pickup along with fewer cracks. The chemical composition of the recast layer was distinguished largely from the base material when using the graphite electrode. However, it was less influenced in the case of the copper electrode.

#### 3.1.3. Surface Morphology of Recast Layers before Tribological Testing

**Figure 7:**
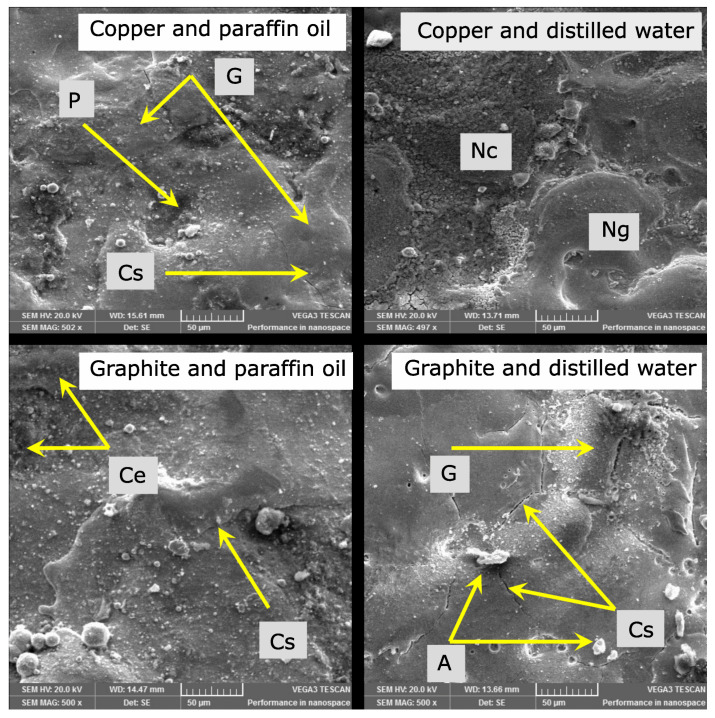
SEM micrographs before the tribo-test at 500× magnification (Cs stands for cracked surface, G for globules, P for pits, A for appendage, Ce for carbon enriched, Nc for no cracks and Ng for no globules).

During surface morphology, surface defects were examined before the tribological test by taking SEM images at 500× magnification as presented in Figure 7. When graphite was used as an electrode material and paraffin oil as a dielectric medium, thicker surface cracks were uniformly distributed on the whole recast layer. Blackish content was collected in the crater-like cavities. These were due to the graphite’s decomposition and more cracking of the paraffin oil, as reported by [38]. The recast layer produced by the graphite electrode with distilled water as a dielectric was patchy, and there were appendages and globules of re-solidified material, along with some cracks. There were surface pits and globules on the surface produced by the combination of the copper electrode and the paraffin oil dielectric. Surface cracks were also seen. As reported by [40], when the magnitude of the residual stresses exceeds the strength of the material, surface cracks will be produced. It was noted that the copper electrode with the distilled water dielectric resulted in a comparatively smoother recast layer with less cavities and no globules. Also, no cracks were visible on or across the surface, albeit with some imperfections and cavities. However, in the tests, these minor imperfections seemed to play little role since its surface roughness was low and tribological performance was comparatively better. The recast layers displayed more surface cracks using paraffin oil as compared to distilled water. This proves that surface cracking is directly dependent upon the amount of carbon content present in the recast layer formed.

#### 3.1.4. Cross-Sectional Investigation of Recast Layers

When copper was used as an electrode and distilled water as a dielectric at a discharge current of 4.5 A, the recast layer thickness was 5.85 μm. The recast layer thickness reached 16 μm with an increase in the discharge current to 15 A. For the Graphite–Distilled water combination, the recast layer thickness increased from 3.5 μm to 10.13 μm. Similarly, for the Copper–Paraffin oil and Graphite–Paraffin oil combinations, the recast layer thickness increased up to 22.13 μm and 21.83 μm, respectively.

For the Distilled water–Copper and Distilled water–Graphite combinations, the thickness of the recast layers increased almost linearly. On the other hand, paraffin oil behaved non-linearly and distinctly for both electrodes and produced a higher recast layer thickness. These observations could be due to the higher thermal conductivity of distilled water as compared to paraffin oil. In the case of distilled water, thermal energy dissipated rapidly and melted a lesser amount of material. In addition, the high discharge current increased the melting and vaporization of the specimen material. Hence, the recast layer thickness directly depends upon the discharge current. Similar trends have also been reported in the literature [41]. The hardness trends of the recast layers are summarized in the Table 7.

When the discharge current varied from 4.5 A to 15 A, the hardness of the recast layers varied from 85 HRB to 117 HRB. The discharge current seems to have little effect on the hardness of the recast layers. In addition, the thickness of the recast layers had no effect on their hardness. These observations are consistent with the findings in [42].

### 3.2. Tribological Performance of Recast Layers

The effect of variations in EDM parameters on the tribological performance was observed by the tribo-tests. Average wear depth, coefficient of friction, friction force and contact surface temperature were measured at the sliding speed of 6.28 m/s, applied load of 25 N and test duration of 10 min. Tribological testing was performed under oil lubricated conditions. It was observed that the specimen surface and the counter–disk interaction became more severe, and load was carried totally by these during testing. The coefficient of friction was high, along with significant wear. It can be concluded that the lubrication regime was boundary lubrication. In the boundary lubrication regime, the physical properties of the lubricant such as density and viscosity are not as important as the properties of the surfaces in contact [43]. Therefore, the worn surface characteristics of recast layers being presented are mainly due to the differences in surface integrity. The contact point of the test specimen was fixed, but it kept changing for the counter-disk in each revolution. The experiments were carried out by variations in EDM parameters followed by tribo-tests. Thus, the tribology of the recast layers was characterized and presented in the forthcoming sections.

#### 3.2.1. Friction Force

**Figure 8:**
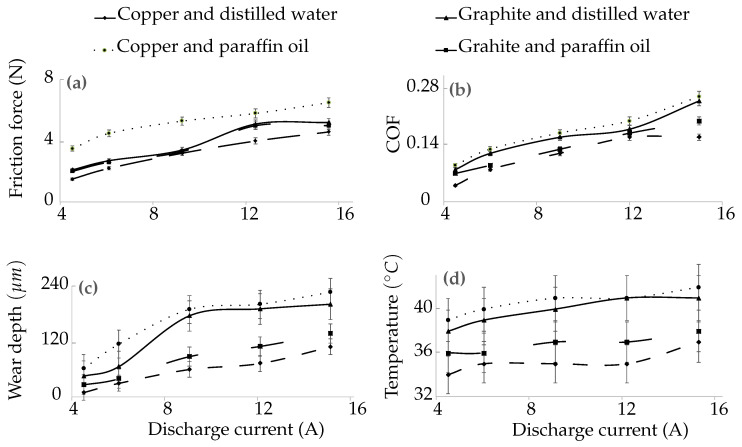
Tribological performance for test of 10 min duration (**a**) friction force (for first 5 min), (**b**) coefficient of friction (for last 5 min), (**c**) wear depth (for 10 min) and (**d**) surface temperature (for 10 min) versus discharge current.

A beam type load cell (Figure 3) measured the frictional force, which is displayed on PC. It was found that friction force increased with the discharge current and varied from 1.5 N to 6.5 N. The copper and paraffin oil combination produced the highest friction force (6.5 N at 15 A). It could be attributed to the surface defects as observed during SEM. The copper and distilled water combination had the least friction force (4.6 N at 15 A), as presented in Figure 8a and Table 8. The reason for this observation was its smooth surface with the least surface defects and $R_{a}$ value. The friction force by Graphite–Distilled water (5.2 N at 15 A) and Graphite–Paraffin oil (5 N at 15 A) was in between the combinations mentioned above. At a high discharge current, the high discharge energy produced rough surfaces, contributing to the higher friction force. Moreover, the enriched carbon content and surface defects increased the friction force.

#### 3.2.2. Coefficient of Friction

The friction coefficient kept increasing with the increase in the discharge current (0.04–0.26) in all cases. The copper and paraffin oil combination had the highest COF value (0.26 at 15 A), followed by Graphite–Distilled water (0.25 at 15 A) and Graphite–Paraffin oil (0.2 at 15 A). The copper and distilled water combination exhibited the lowest COF (0.16 at 15 A), as shown in Figure 8b and Table 8. As the coefficient of friction is the ratio of the friction force and the applied load, these observations were due to similar reasons as explained for the friction force.

#### 3.2.3. Wear Depth

LVDT was used for sensing the wear rate. The movement of the plunger (Figure 3) is displayed as wear on the controller. The wear depth was found to increase with discharge current and ranged from 9 µm to 226 µm. The copper and paraffin oil combination produced the highest wear depth (226 µm at 15 A) due to the surface pits, globules and surface cracks produced during EDM. The Graphite–Distilled water and Graphite–Paraffin oil combinations had wear depths of 200 µm and 137 µm at 15 A, respectively. The copper and distilled water combination exhibited the least wear depth value (108 µm at 15 A) due to the smooth surface with the least $R_{a}$ value and no surface cracks, as shown in Figure 8c and Table 8. At high discharge current, larger and deeper craters were generated, resulting in increased wear depth. These outcomes were also supported by the studies of [36,37]. The recast layer with the combination of copper and distilled water exhibited 43% better wear resistance when compared with Graphite–Paraffin oil, 143% better than Graphite–Distilled water and 185% better than Copper–Paraffin oil. So, the best wear-resistant recast layer was due to the least roughness value and no surface defects. At high discharge current, larger and deeper craters were generated, resulting in increased wear depth.

#### 3.2.4. Contact Surface Temperature

The contact surface temperature was measured by a k-type thermocouple (Figure 3). The terminal ends of the thermocouple were connected to the machine panel and from there to the controller back panel. The contact surface temperature, as shown in Figure 8d and Table 8, also increased with the discharge current (34–42 °C) for all combinations of electrodes and dielectrics. The copper with paraffin oil combination produced the highest contact surface temperature (42 °C at 15 A) followed by the Graphite–Distilled water (41 °C at 15 A) and Graphite–Paraffin oil (38 °C at 15 A) combinations. The copper and distilled water combination produced the least contact surface temperature (37 °C at 15 A). An increase in temperature softened the material and resulted in the wear of the recast layer. Thus, the wear depth and contact surface temperature by the combination of the copper electrode and paraffin oil dielectric were increased. The contact surface temperature increased due to the increase int the surface roughness, surface defects and diffused carbon content in the recast layers.

#### 3.2.5. Weight Loss

**Figure 9:**
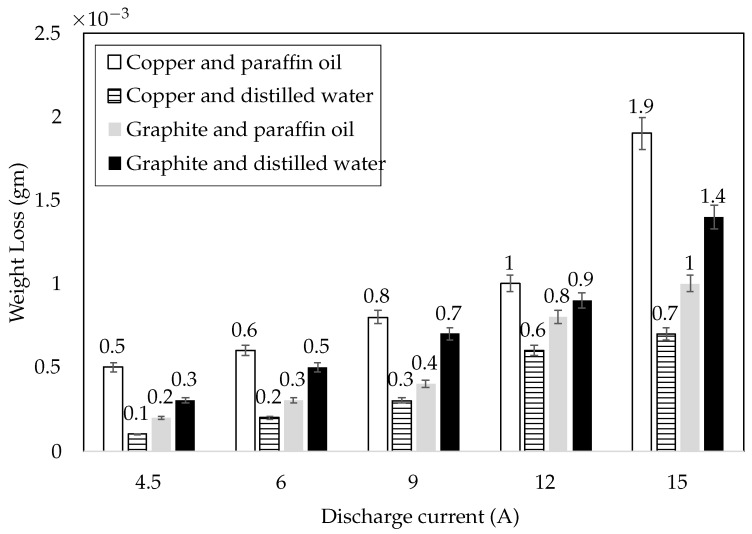
Weight loss of the specimens against discharge current (sliding speed of 6.28 m/s, applied load of 25 N and test duration of 10 min).

Specimen masses were measured before and after the tribo-tests, and their difference was the actual weight loss. It was determined that with the increase in the discharge current, the weight loss increased (0.0001 g to 0.0019 g). The copper electrode with paraffin oil dielectric gave the maximum weight loss (0.0019 g at 15 A). The Graphite–Distilled water and Graphite–Paraffin oil combinations had 0.0014 g and 0.001 g (at 15 A) weight losses, respectively. The copper and distilled water combination gave minimum weight loss (0.0007 g at 15 A), as shown in Figure 9. The recast layer with the combination of graphite and paraffin oil exhibited 42% more weight loss when compared with Copper–Distilled water. Similarly, Graphite–Distilled water and Copper–Paraffin oil had 100% and 185% greater weight loss, respectively, as compared to Copper–Distilled water.

At elevated temperature, micro-thermal softening occurred and reduced the bond strength of the recast layer. More weight loss has good agreement with the similar phenomenon. The results evidenced that at all values of the discharge current, weight loss was directly influenced by surface roughness, chemical composition and surface defects. Surface defects majorly contributed to significant weight loss.

**Figure 10:**
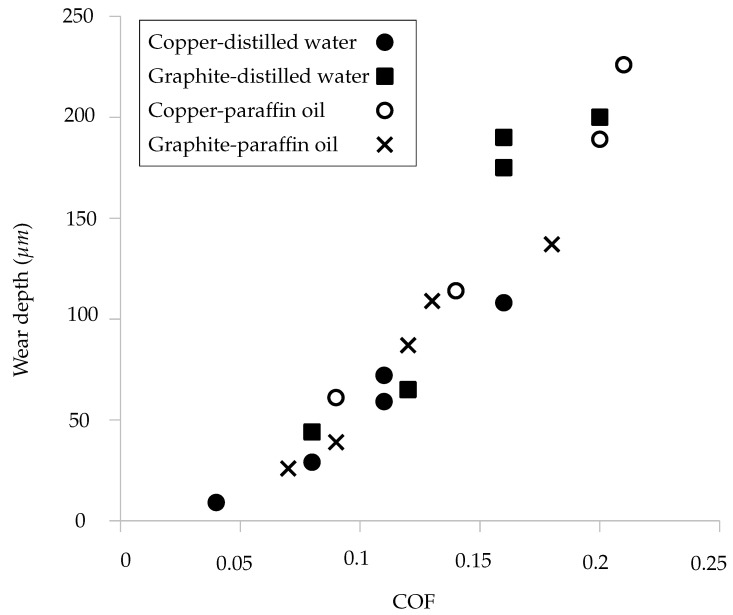
Relationship between coefficient of friction and wear depth.

Although tribological performance was analyzed with the variations in the EDM parameters, a characterization was made with the relationship between wear depth and the coefficient of friction, which has been shown in Figure 10. It was observed that there is a direct relation between these, which is in accordance with the wear energy formulation [44].

There was a limitation in the tribotester that when the wear progressed, the loading lever tended to subtend an angle that caused a variation in the contact area. However, this limitation was compensated for by the fact that all the specimens were subjected to the same conditions. This observation allowed for at least a qualitative comparison of the tribological performance of all the samples, if not quantitative.

#### 3.2.6. Surface Morphology of Recast Layers after Tribological Testing

**Figure 11:**
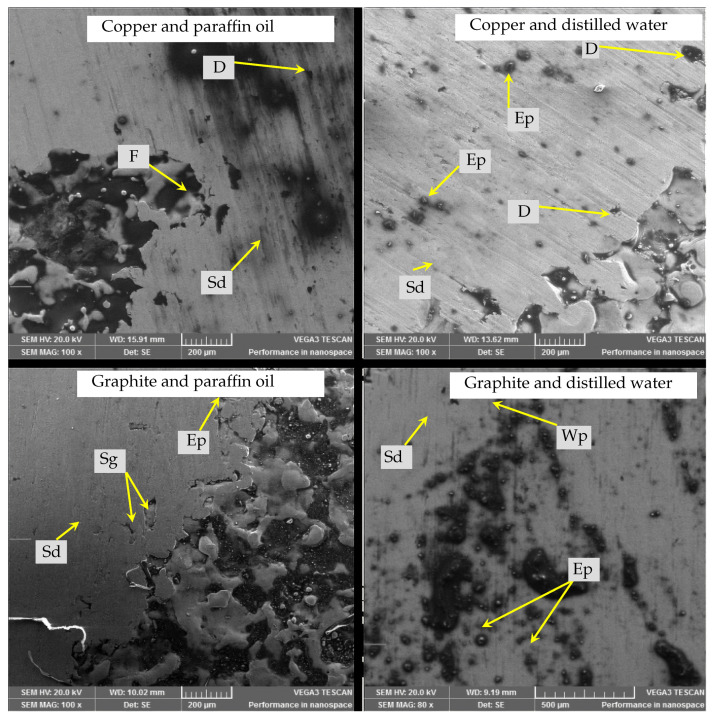
SEM micrographs after the tribo-test at 500× magnification (D stands for debris, F for fracture, Sg for shallow grooves, Wp for wear pit, Ep for embedded particle and Sd for sliding direction).

Micrographs of recast layers under an applied load of 25 N, sliding distance of 3770 m and velocity of 6.28 m/s are presented in Figure 11.

During the worn surface study, severe wear with fractures and debris was observed in the case of the recast layer formed by the copper electrode and paraffin oil dielectric. This was because of the high shear force applied at the specimen surface during test. Dark impressions filled with high carbon were found, and the recast layer was partially removed. The observation was also supported by the significant weight loss of the specimen. Using a graphite electrode with distilled water as a dielectric, wear pits and embedded particles were observed on the recast layer. Trenches remained, though surface removal was visible. The reason for this observation was surface defects as observed before the tribological test. With the graphite electrode and paraffin oil as a dielectric, the recast layer was quite smooth, and there were tiny impressions before the tribo-test, which were removed during testing, and large trenches were unaffected. Shallow grooves and embedded particles were also found in this case because of the thicker surface cracks. As the recast layer had the highest contamination of carbon content, it offered more resistance to abrasion. Minimal weight loss during testing also supported this phenomenon. It was observed that the copper electrode with a distilled water dielectric resulted in the slight removal of ridges. Wear particles were pressed into the cavities. Some embedded particles were also sparsely found on the smooth recast layer; hence, it resulted in the least weight loss among all the combinations. SEM micrographs showed largely abrasive wear mechanism at play during the test.

## 4. Conclusions

This work investigates the effect of EDM parameters—discharge current, electrodes and dielectric media—on the tribological performance of the recast layers of AISI 304L. Moreover, this research presents the possible enhancement in tribological performance by a proper combination of EDM parameters, a condition that is not sufficiently addressed in the literature. The effects of the variations in these parameters in the boundary lubrication regime are summarized below:

- Tribological performance is directly influenced by the discharge current during EDM, regardless of the electrodes and dielectric media. The coefficient of friction, contact surface temperature, wear depth, weight loss and surface roughness increase with the discharge current.
- The recast layer formed by the copper electrode with a distilled water dielectric exhibits the best tribological performance – in terms of wear depth among all the combinations: 43% better than Graphite–Paraffin oil, 143% better than Graphite–Distilled water and 185% better than Copper–Paraffin oil. On the contrary, the recast layer formed by the copper electrode with a paraffin oil dielectric has the poorest tribological performance. The combinations of Graphite–Paraffin oil and Graphite–Distilled water behave intermediately.
- EDM alters the surface roughness, chemical composition and surface morphology of the resulting recast layers. The impact of the surface defects is more significant on tribological performance than surface roughness and chemical composition.
- Graphite electrode and paraffin oil dielectric produce the roughest recast layer with more surface cracks and the contamination of carbon content. On the other hand, the copper electrode and distilled water dielectric combination exhibit the smoothest recast layer with no cracks.
- Recast layers formed by combinations of Copper–Distilled water and Copper–Paraffin oil show the best and the poorest surface characteristics, respectively, in terms of surface defects.

Experimental evidences emphasize that different control parameters of EDM significantly dictated the tribological behavior of produced recast layers. Therefore, it is important to thoroughly analyze these parameters to economically achieve the desired surface properties during the machining process. 

## Figures and Tables

**Table 1:** Composition analysis of the specimen.

Element	%Weight
C	0.02
Si	0.57
Mn	1.70
P	0.02
S	0.01
Cr	17.3
Al	0.03
Co	0.05
Cu	0.06
Nb	0.01
Ti	0.01
V	0.06
Mo	2.60
Ni	14.5
W	0.03
Fe	63.1

**Table 2:** Experimental conditions for EDM.

Work Piece	AISI 304L	
Diameter	8	mm
Length	25	mm
Dielectrics	Paraffin oil and distilled water	
Discharge current	4.5, 6, 9, 12 and 15	A
Down time	5	µs
Up time	5	µs
Electrodes	Copper and Graphite	
Gap	0.125	mm
Pulse duration	90	µs
Pulse off time	5	µs

**Table 3:** Composition analysis of the counter-disk.

Element	%Weight
C	1.14
Si	0.18
Mn	0.38
P	0.05
S	0.04
Cr	1.25
Al	0.02
Co	0.01
Cu	0.12
Nb	0.01
Ti	0.01
V	0.01
Mo	0.01
Ni	0.07
W	0.01
Fe	96.7

**Table 4:** Properties of SuperMatic 4T SAE 10W-40 engine oil.

Viscosity (40 °C)	100	mm^2^/s
Viscosity index	154	
Sulfated ash	0.99	mass %
Phosphorus	0.1	mass %

**Table 5:** Experimental conditions for tribological testing.

Work Piece	AISI 304L	
Diameter	8	mm
Length	25	mm
Temperature	25	°C
Humidity	RH 47	%
Applied Load	25	N
Test duration	10	min
Rotational speed	1000	rpm
Track radius	60	mm
Sliding distance	3770	m
Sliding speed	6.28	m/s

**Table 6:** Surface roughness for electrode–dielectric combinations of Copper–Paraffin oil (CO), Copper–Distilled water (CW), Graphite–Distilled water (GW) and Graphite–Paraffin oil (GO).

Current	CO (Ra)	CW (Ra)	GW (Ra)	GO (Ra)
4.5	3.607	2.987	3.509	3.745
6	4.610	3.709	3.830	4.766
9	4.842	4.201	4.479	5.163
12	6.230	4.400	5.281	7.035
15	8.918	4.849	8.719	11.184

**Table 7:** Recast layers hardness for electrode–dielectric combinations of Copper–Paraffin oil (CO), Copper–Distilled water (CW), Graphite–Distilled water (GW) and Graphite–Paraffin oil (GO).

Current (A)	CO (HRB)	CW (HRB)	GW (HRB)	GO (HRB)
4.5–15	85–115	94–109	91–116	101–117

**Table 8:** Tribological performance for electrode–dielectric combinations of Copper–Paraffin oil (CO), Copper–Distilled water (CW), Graphite–Distilled water (GW) and Graphite–Paraffin oil (GO).

	Friction Force, N				COF			
**Current (A)**	**CW**	**GW**	**CO**	**GO**	**CW**	**GW**	**CO**	**GO**
4.5	1.5	2.1	3.5	2	0.04	0.08	0.09	0.07
6	2.2	2.7	4.5	2.6	0.08	0.12	0.13	0.09
9	3.2	3.4	5.3	3.3	0.12	0.16	0.17	0.13
12	4	5.1	5.8	5	0.16	0.18	0.2	0.17
15	4.6	5.2	6.5	5	0.16	0.25	0.26	0.2
	**Wear depth, µm**				**Temperature °C**			
4.5	9	44	61	26	34	38	39	36
6	29	65	114	39	35	39	40	36
9	59	175	189	87	35	40	41	37
12	72	190	200	109	35	41	41	37
15	108	200	226	137	37	41	42	38

## Data Availability

The data used to support the findings of this study are included within the article.
